# Near-Infrared Light-Responsive Shell-Crosslinked Micelles of Poly(d,l-lactide)-*b*-poly((furfuryl methacrylate)-*co*-(*N*-acryloylmorpholine)) Prepared by Diels–Alder Reaction for the Triggered Release of Doxorubicin

**DOI:** 10.3390/ma14247913

**Published:** 2021-12-20

**Authors:** Sonyabapu Yadav, Kalyan Ramesh, Parveen Kumar, Sung-Han Jo, Seong II Yoo, Yeong-Soon Gal, Sang-Hyug Park, Kwon Taek Lim

**Affiliations:** 1 Department of Display Engineering, Pukyong National University, Busan 48513, Korea; yadav555sy@gmail.com (S.Y.); kramesh.chem@gmail.com (K.R.); parveenkmr19@gmail.com (P.K.); 2 Department of Chemistry, University of Massachusetts Lowell, Lowell, MA 01854, USA; 3 Department of Biomedical Engineering, Pukyong National University, Busan 48513, Korea; josunghan91@gmail.com (S.-H.J.); shpark1@pknu.ac.kr (S.-H.P.); 4 Department of Polymer Engineering, Pukyong National University, Busan 48513, Korea; siyoo@pknu.ac.kr; 5 Department of Fire Safety, Kyungil University, Gyeongsan 38428, Korea; ysgal@kiu.ac.kr

**Keywords:** shell-crosslinked micelles, RAFT polymerization, Diels–Alder reaction, NIR light-responsive

## Abstract

In the present study, we developed near-infrared (NIR)-responsive shell-crosslinked (SCL) micelles using the Diels–Alder (DA) click reaction between an amphiphilic copolymer poly(d,l-lactide)_20_-*b*-poly((furfuryl methacrylate)_10_-*co*-(*N*-acryloylmorpholine)_78_) (PLA_20_-*b*-P(FMA_10_-*co*-NAM_78_)) and a diselenide-containing crosslinker, bis(maleimidoethyl) 3,3′-diselanediyldipropionoate (BMEDSeDP). The PLA_20_-*b*-P(FMA_10_-*co*-NAM_78_) copolymer was synthesized by RAFT polymerization of FMA and NAM using a PLA_20_-macro-chain transfer agent (PLA_20_-CTA). The DA reaction between BMEDSeDP and the furfuryl moieties in the copolymeric micelles in water resulted in the formation of SCL micelles. The SCL micelles were analyzed by ^1^H-NMR, FE-SEM, and DLS. An anticancer drug, doxorubicin (DOX), and an NIR sensitizer, indocyanine green (ICG), were effectively incorporated into the SCL micelles during the crosslinking reaction. The DOX/ICG-loaded SCL micelles showed pH- and NIR-responsive drug release, where burst release was observed under NIR laser irradiation. The in vitro cytotoxicity analysis demonstrated that the SCL was not cytotoxic against normal HFF-1 cells, while DOX/ICG-loaded SCL micelles exhibited significant antitumor activity toward HeLa cells. Thus, the SCL micelles of PLA_20_-*b*-P(FMA_10_-*co*-NAM_78_) can be used as a potential delivery vehicle for the controlled drug release in cancer therapy.

## 1. Introduction

Polymeric self-assemblies such as vesicles and micelles prepared from amphiphilic copolymers have shown encouraging results as chemotherapeutic nanocarriers due to their distinct shell–core nanostructures [1,2]. The hydrophobic blocks of micellar cores can entrap and solubilize hydrophobic drugs [3,4]. In addition, hydrophilic coronas can efficiently protect the hydrophobic core and their entrapped therapeutics from the external biological environment during blood circulation to diminish the nonspecific adsorption by protein [5,6,7]. However, there are still several concerns associated with self-assembled polymeric micelles such as stability after drug encapsulation and structural integrity, limiting their use as promising nanovehicles for therapeutics [8,9]. Polymeric micelles loaded with therapeutics face high dilution and shear stress after intravenous injection [10,11]. Consequently, the micelles dissociate on reaching their concentration below critical micelle concentration (CMC), leading to premature release of therapeutics before arriving at their targets [12]. This dissociation shortens the nanoformulation shelf-life, resulting in reduced efficacy and various undesirable side-effects [13]. Moreover, physical encapsulation of therapeutics within the micelles inevitability leads to their premature release during storage, resulting in shortened shelf-life of the nanoformulation. The core/shell-crosslinking (CCL/SCL) approach has been developed to rise to the challenges and to achieve effective delivery of therapeutics even if the micellar concentration is reduced in blood circulation [14,15,16,17]. Shell-crosslinking has received great attention since Wooley et al. first introduced SCL micelles [18,19]. Most of the recent efforts have focused on fabrication of SCL micelles having hollow cores and tunable hydrophilic/hydrophobic cores. In principle, SCL micelles having a hydrophilic/hydrophobic core offer an advantage over hollow cores because no core removal step is needed, and they also provide the potential for the triggered release of therapeutics upon different chemical stimuli. Hu et al. reported the SCL micelles of well-defined amphiphilic diblock copolymers (PCL-*b*-P(OEGMA-*co*-MAEBA) [20]. These micelles were subsequently shell-crosslinked with dithiol bis(propanoic dihydrazide) to induce acylhydrazone crosslinking with p-(methacryloxyethoxy)benzaldehyde (MAEBA) moieties. Ding et al. prepared the SCL micelles of diblock copolymer, polystyrene-*b*-poly(2-cinnamoylethyl methacrylate) (PS-*b*-PCEMA) [21]. The shell-crosslinking took place through the photoinduced cycloaddition of CEMA. 

Typically, end-terminal bifunctional spacers have been utilized to crosslink micelles by coupling with the reactive pendant groups of polymeric chains. Various methods have been utilized to prepare SCL micelles including acylhydrazone crosslinking [20,22], photo-induced crosslinking [21], diamide crosslinking [23], and carbodiimide coupling [24]. Recently, a crosslinking reaction via click chemistry has attracted great attention due to its various advantages such as simple reaction conditions, high yield, and inoffensive byproducts. Click reactions include copper-catalyzed alkyne cycloaddition, Diels–Alder (DA) cycloaddition, thiol-Michael addition, and thiol-ene click reaction [25]. Among them, the DA reaction has the most potential due to many advantages including high yield, catalyst-free, benign solvents, simple reaction conditions, and no side product [26]. Various polymeric CCL micelles of amphiphilic block copolymers using DA reaction have been reported recently. Bapat et al. synthesized CCL micelles using a DA reaction between the furan functional blocks of well-defined block copolymers and bismaleimide crosslinkers, and the thermoreversibility of DA linkages within the cores was studied [27]. However, to the best of our knowledge, there have so far been no studies on SCL micelle preparation using DA reaction. 

Biodegradable and biocompatible amphiphilic block copolymers have shown immense potential in drug delivery applications [28]. In this work, we designed an amphiphilic copolymer composed of three different monomers with desirable traits, namely, d,l-lactide (LA), *N*-acryloylmorpholine (NAM), and furfuryl methacrylate (FMA). PLA forms an effective biocompatible and biodegradable hydrophobic core of amphiphilic block copolymers for hydrophobic drugs encapsulation. PNAM is a promising hydrophilic polymer that has been utilized to develop drug delivery carriers due to its exceptional biological and physiological properties such as low cellular toxicity, biocompatibility, blood compatibility, and high water solubility. Moreover, the ability of furan moieties in PFMA to cause DA cycloaddition reaction with dienophiles such as maleimide provides a valuable role to handle the functionalization of copolymers. Thus, the introduction of a small amount of FMA into the hydrophilic portion of the copolymer could provide the means to crosslink the shells of the micelles via the facile DA reaction.

To achieve the maximum benefit, some chemical modifications have been introduced to the crosslinked structure, which could trigger the release of therapeutics in response to internal stimuli such as the targeted tumor microenvironment [29,30,31] or external stimuli such as magnetic field, light (near-infrared (NIR) or ultraviolet (UV)), ultrasound, and electric field [32,33]. Light is the most prominent external stimulus to trigger the release of encapsulated therapeutics due to its noncontact application, which improves the patient’s compliance [34,35]. UV (high-frequency light, 100–400 nm) and NIR (low-frequency light, 650–900 nm) have been utilized in recent years as external stimuli for drug delivery applications [36]. NIR light is sufficient to induce the biochemical response and is considered to be safer compared to UV light due to low radiation energy [37,38,39,40]. Our group recently reported NIR-responsive CCL micelles of poly(ethylene oxide)-*b*-poly(furfuryl methacrylate) diblock copolymers containing indocyanine green (ICG), an FDA-approved NIR fluorescent dye [41]. The diblock copolymers were core-crosslinked with a diselenide-containing crosslinker through the DA click reaction. NIR irradiation induced the decrosslinking of CCL micelles by triggering ICG to produce reactive oxygen species (ROS) which subsequently decomposed the diselenide bonds in the crosslinks. The decrosslinking can be easily controlled to achieve the desired release of entrapped drugs inside micelles; thus, SCL micelles which exhibit triggered drug release in response to external stimuli, especially NIR light, are also highly desirable.

Doxorubicin (DOX) is an anthracycline antibiotic, isolated from *Streptomyces peucetius*, having proficiency in the treatment of various hematological and solid tumors [42,43]. The three major anticancer mechanisms of DOX include inhibiting DNA topoisomerase II activity, DNA intercalation, and enhancing production of free radicals (ROS) [44]. However, the benefits of DOX are limited by major obstacles such as the development of resistance and dose-dependent toxicity [45]. The improvement of DOX efficacy in cancer cells has been documented by eliminating these obstacles using nanocarriers for its delivery [42].

**Scheme 1:**
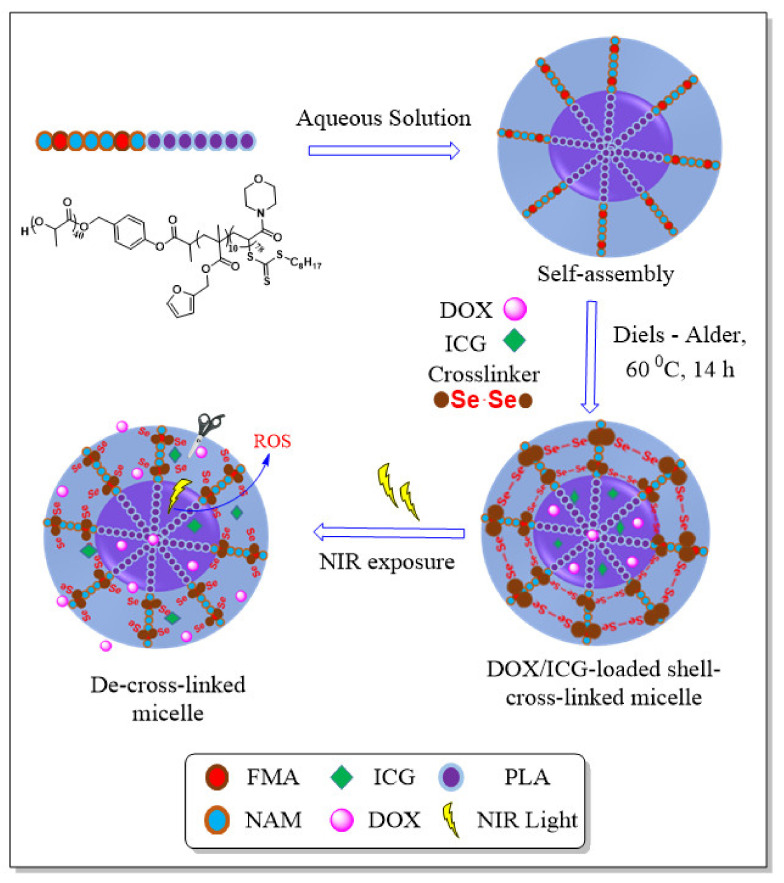
Preparation of SCL micelles of PLA_20_-*b*-P(FMA_10_-*co*-NAM_78_) and DOX release under NIR exposure.

Herein, we prepared novel poly(d,l-lactide)_20_-*b*-(poly(furfuryl methacrylate)_10_-*co*-poly(*N*-acryloylmorpholine)_78_), (PLA_20_-*b*-P(FMA_10_-*co*-NAM_78_)) copolymers via addition-fragmentation chain-transfer (RAFT) copolymerization of FMA and NAM using a PLA_20_-macro-chain transfer agent (PLA_20_-CTA) [22,46,47,48]. The micelles were formed in an aqueous medium, which were composed of the core of the hydrophobic moiety (PLA) and the shell of the hydrophilic moiety PNAM having a small amount of PFMA. The shell-crosslinking was induced by the DA reaction between the furfuryl group of PFMA and the maleimide group of the crosslinker, bis(maleimidoethyl) 3,3′-diselanediyldipropionoate (BMEDSeDP), producing NIR-responsive micelles (Scheme 1). DOX and ICG were simultaneously incorporated into the micelles. The resulting SCL micelles controlled the DOX release under physiological environment but rapidly released the DOX under NIR irradiation. The micelles were biocompatible with normal cells and exhibited cytotoxicity to the HeLa cancer cells, which was further enhanced upon NIR irradiation. Thus, the PLA_20_-*b*-P(FMA_10_-*co*-NAM_78_)-based SCL micelles could be a potential candidate to be used as nanocarriers for on-demand NIR responsive platforms in cancer therapy [49].

## 2. Materials and Methods

### 2.1. Materials

Selenium powder (99.99%), 4-(dimethyl amino) pyridine (DMAP, 99%), 3-bromopropionic acid (97%), furan (99%), *N*,*N*′-dicyclohexylcarbodiimide (DCC, 99%), maleic anhydride (99%), triethylamine, ethanol (98%), fetal bovine serum (FBS), benzoic acid, dimethyl formamide (DMF), AIBN (98%), furfuryl methacrylate (FMA, 97%), and 1, 8-diazabicyclo [5.4.0] undec-7-ene (DBU, 98%) were purchased from Sigma-Aldrich (Seoul, Korea). d,l-Lactide (LA, 99.0%) and *N*-acryloylmorpholine (NAM, 98.0%) were purchased from Sigma-Aldrich (Seoul, Korea), and used after purification. DOX hydrochloride (DOX⋅HCl) was received from Boryung Pharm. Co. (Seoul, Korea). Ethanolamine (99%) was received from Fluka (Seoul, Korea). The cell viability assay solution (EZ-Cytox, WST-1^®^) was purchased from Daeil Lab Services (Seoul, Korea). Dichloromethane (DCM), ethyl acetate (EtOAc), dimethyl formamide (DMF), and tetrahydrofuran (THF) were obtained from Duksan (Busan, Korea) and used without further purification unless otherwise mentioned.

### 2.2. Characterization 

The morphology of samples was investigated by means of a field-emission scanning electron microscope (FE-SEM) equipped with an energy-dispersive X-ray spectrometer (Hitachi JEOL-JSM-5 6700F system, Tokyo, Japan). Dynamic light scattering (DLS) measurements were performed using an electrophoretic light scattering instrument (Zetasizer Nano ZS, Malvern Panalytical Ltd, Malvern, UK), equipped with a He–Ne laser and ELS controller at a wavelength of 633 nm, and the scattering light intensity was detected at 90° to an incident beam. UV/Vis spectra were recorded on a UV/Vis spectrophotometer (Optizen POP, Daejeon, Korea). The molecular weight distribution (*Ð*) and number average molecular weight (M_n_) were determined at 25 °C using a gel permeation chromatograph (GPC: Agilent Technologies, USA) equipped with an RID detector, PL gel column (5 μm; 102–104 Å), and HP 1100 pump. THF was used as an eluent (flow rate: 1 mL/min), and a calibration curve was constructed using polystyrene standards. NMR spectra were recorded on a JNM-ECP 400 MHz (JEOL, Akishima-shi, Japan) NMR spectrometer. 

#### 2.2.1. Synthesis of the BMEDSeDP Crosslinker

1-(2-Hydroxyethyl)-pyrrole-2, 5-dione (HEMI) and 3, 3′-diselanediyldipropionic acid (DSeDPA) were prepared according to the procedure reported earlier [41]. DMAP (24 mg, 0.2 equiv.) and DCC (472 mg, 1.7 equiv.) were added to a stirred solution of HEMI (588 mg, 2 equiv.) in 6 mL of THF at 0 °C, followed by the addition of DSeDPA (640 mg, 1 equiv.) in 10 mL of THF. The mixture was stirred for 30 min at 0 °C and further stirred at room temperature (RT) for 24 h. The precipitated dicyclohexylurea as a byproduct was filtered off. The filtrate was concentrated and extracted with DCM, and the combined organic layers were washed with a saturated aq. NaCl solution, dried over anhydrous NaSO_4_, and filtered. The solvent was evaporated, and the residue was purified using column chromatography (50% EtOAc/hexane) to get a white solid of BMEDSeDP (980 mg, 84%). ^1^H-NMR (400 MHz, CDCl_3_): *δ* = 2.78 (t, *J* = 7.2 Hz, 4H), 3.05 (t, *J* = 7.2 Hz, 4H), 3.82–3.77 (m, 4H), 4.26 (t, *J* = 4.5 Hz, 4H), 6.73 (s, 4H) ppm.

#### 2.2.2. Synthesis of 4-(Hydroxymethyl)phenyl-2-propanoate 2-Octyl-trithiocarbonate (HMPPOT)

HMPPOT was synthesized in two steps. In the first step, 4-hydroxy benzyl alcohol (5 g, 1 equiv.) and trimethylamine (6.9 mL, 1.2 equiv.) were dissolved in dry THF (200 mL) with constant stirring under N_2_ atmosphere. 2-Chloropropionyl chloride (3.9 mL, 1 equiv.) was added dropwise to the above solution at 0 °C and stirred for 48 h at RT. Residues from the reaction were removed by filtration, and the filtrate was evaporated. The resulting solid was purified by column chromatography to obtain 4-(hydroxymethyl)phenyl 2-chloropropanoate as a white solid (6.5 g, 75%). ^1^H-NMR (400 MHz, CDCl_3_): *δ* = 1.3 (d, 3H), 4.45 (s, 2H), 4.6 (q, 1H), 6.9 and 7.23 (dd, 4H) ppm.

In the second step, 4-(hydroxymethyl)phenyl 2-chloropropanoate (3.5 g, 1 equiv.) dissolved in acetone (40 mL) was added dropwise to the solution of potassium octyl-trithiocarbonate (5.2 g, 1.2 equiv.) in acetone (200 mL), and the mixture was stirred for 14 h at RT. The precipitated byproduct was removed by filtration, and the filtrate was evaporated. The residue was dissolved in ethyl acetate and washed with water (3 × 150 mL), dried over anhydrous NaSO_4_, filtered, and evaporated. The product was purified by column chromatography to obtain HMPPOT as a pale-yellow solid (5.5 g, 80%). ^1^H-NMR (400 MHz, CDCl_3_): *δ* = 1.7 (d, 3H), 4.65 (s, 2H), 4.9 (q, 1H), 7.1–7.7 (dd, 4H), 3.4 (d, 2H), 0.9 (t, 3H), 1.2–1.5 (m, 12H) ppm.

#### 2.2.3. Synthesis of PLA_20_-Chain Transfer Agent (PLA_20_-CTA)

The synthesis of PLA_20_-CTA was carried out via ring-opening copolymerization (ROP) of LA using a dual initiator, HMPPOT, and a catalyst, 1, 8-diazabicycloundec-7-ene (DBU). In brief, under N_2_ atmosphere in a glove box, LA (2.0 g, 26 equiv.) was dissolved in 8.0 mL of dry DCM, and then HMPPOT (200 mg, 1 equiv.) and DBU (40 μL, 42.2 mg, 0.5 equiv.) were added to the mixture and stirred for 20 min at RT to carry out the polymerization. After 20 min, benzoic acid (39 mg) was added to stop the polymerization. The resulting PLA_20_-CTA was purified by precipitating in cold CH_3_OH and dried under vacuum to get the product as a white solid (2.05 g, 92%). ^1^H-NMR (400 MHz, CDCl_3_): *δ* = 7.08 (d, 2H), 7.33 (d, 2H), 5.06 (d, 2H), 4.36 (q, 1H), 3.39 (m, 2H), 1.71 (d, 3H), 0.87 (m, 3H) ppm. 

#### 2.2.4. Synthesis of PLA_20_-*b*-P(FMA_10_-*co*-NAM_78_) 

PLA_20_-CTA (100 mg, 1 equiv.), FMA (98 mg, 20 equiv.), NAM (332 mg, 80 equiv.), and AIBN (1 mg, 0.2 equiv.) were dissolved in dry 1, 4-dioxane (4 mL) and purged with N_2_ for 40 min. The polymerization was carried out at 70 °C for 12 h, and then the solution was frozen with liquid nitrogen to stop the polymerization. The polymer was precipitated twice in cold diethyl ether and dried under vacuum to get the PLA_20_-*b*-P(FMA_10_-*co*-NAM_78_) copolymer as a white solid (Yield = 400 mg, 80%). ^1^H-NMR (400 MHz, CDCl_3_): *δ* = 7.45 (s, 1H), 6.36 (d, 2H), 5.02 (m, 2H), 1.24 (m, 3H), 0.96 (s, 1H) ppm.

#### 2.2.5. Critical Micelle Concentration (CMC) Determination

Several aqueous concentrations of PLA_20_-*b*-P(FMA_10_-*co*-NAM_78_) (0.0005–1.0 mg/mL) were prepared using deionized water. A stock solution of pyrene in acetone was transferred to all the copolymer concentrations to get a final pyrene concentration of 6 × 10^−7^ M in each sample, and acetone was evaporated under nitrogen bubbling. The excitation spectra of all the samples (300–360 nm) were recorded at 394 nm emission wavelength using 5 nm slit width. The excitation spectrum peak intensity ratio of pyrene at 337 and 333 nm (I_337_/I_333_) was plotted against the function of copolymer concentration. CMC value was determined considering the interception point of both tangent straight lines.

#### 2.2.6. Preparation of SCL Micelles via DA Click Reaction 

The crosslinking reaction of PLA_20_-*b*-P(FMA_10_-*co*-NAM_78_) was conducted by dissolving the copolymer (20 mg, 1 equiv.) and BMEDSeDP crosslinker (2 mg, 1.5 equiv.) in ACN (1 mL), followed by the dropwise addition of H_2_O (5 mL) into the solution under vigorous stirring for 1 h. The micellar solution was subsequently heated at 60 °C for 14 h to induce the crosslinking reaction in the micellar shell.

### 2.3. Loading of DOX/ICG in SCL Micelles of PLA_20_-b-P(FMA_10_-co-NAM_78_)

DOX·HCL (1 eq.) and triethylamine (3 eq.) were dissolved in DMF (1 mL) and stirred for 18 h at RT under dark to neutralize the DOX. To prepare the DOX-encapsulated SCL micelles, PLA_20_-*b*-P(FMA_10_-*co*-NAM_78_) (20 mg, 1 equiv.), BMEDSeDP (2 mg, 1.5 equiv.), DOX (3.5 mg in 350 μL of DMF), and ICG (1 mg in 100 μL of PBS) were stirred, followed by the dropwise addition of PBS (pH 7.4; 5 mL) under vigorous stirring for 1 h to form DOX/ICG-loaded micelles. The DOX/ICG-loaded micelles were shell-crosslinked by heating at 60 °C for 14 h under stirring and the resulting solution was dialyzed against 1.5 L of distilled H_2_O (MW cutoff, 3500 kDa) for 24 h, replacing with fresh water every 3 h. Finally, the DOX/ICG-loaded SCL micelles were centrifuged at 1500 rpm for 10 min and unloaded DOX and ICG were removed. The amount of DOX/ICG encapsulated into the SCL micelles was determined using UV/visible spectroscopy. DOX or ICG-loading efficiency (LE) and loading content (LC) were calculated using following equations: LC% = (weight of loaded DOX or ICG/total weight of DOX or ICG-loaded micelles) × 100%; LE% = (weight of loaded DOX or ICG/weight of DOX or ICG used) × 100%

### 2.4. In Vitro DOX Release Study from SCL Micelles

In vitro DOX release from the DOX-loaded SCL micelles was determined using a dialysis method. The DOX-loaded SCL micelles (3 mL, 1 mg/mL) were placed in 13 kDa dialysis bags, which were immersed in 15 mL buffer solutions (pH 7.4 and 5.0) at 37 °C with a 100 rpm shaking rate. To conduct the NIR-responsive release, samples were subjected to 5 min NIR irradiation (808 nm, 2 W·cm^−2^) at appropriate time intervals. The dialysate (1 mL) was withdrawn from every sample for analysis, and the same volume of fresh buffer solution was added. The percentage of DOX release was determined by a cumulative method using UV/Vis absorbance at 485 nm. 

### 2.5. Cell Viability 

HeLa (human cervical cancer, ATCC) and noncancerous HFF-1 (human foreskin fibroblast, ATCC) cells were used for cytotoxicity evaluation. The cells were cultured and maintained in their respective media (HeLa: RPMI (Gibco) + AA (Gibco) + 10% FBS (Gibco); HFF-1: DMEM (Gibco) + AA (Gibco) + 15% FBS (Gibco)), and then incubated under CO_2_ humidified atmosphere (5%) at 37 °C. The cell viability assay was conducted to find out the biocompatibility effect of non-SCL and SCL micelles on HFF-1 normal cells and the cytotoxic effect of DOX-loaded SCL micelles on HeLa cancer cells. Briefly, HeLa and HFF-1 cells were seeded at the density of 2.0 × 10^4^ cells/well and incubated under the same conditions. HFF-1 normal cells were treated with several SCL micelle concentrations (50–500 μg/mL) and HeLa cells were treated with different DOX-loaded SCL micelle and free DOX concentrations (5–80 μg/mL of DOX concentration) and again incubated for 24 h under the same conditions. The WST assay was used (according to manufacturer’s protocol) to determine the cytotoxicity assay.

### 2.6. Confocal Microscopy

The HeLa cells (5.0 × 10^4^ cells) were cultured on sterile coverslips placed in the confocal dish and treated with DOX-loaded SCL micelles and free DOX (40 μg/mL DOX concentration) and incubated for 24 h. The culture medium was removed; HeLa cells were washed with PBS solution thrice to remove the remaining free DOX and DOX-loaded SCL micelles and fixed with 4% paraformaldehyde/PBS solution for 15 min. The cells were again rinsed once with PBS solution, stained by a DAPI/PBS (1 mg/mL) solution, and incubated for 37 °C for 10 min before observing the nuclear morphology of HeLa cells using a fluorescence confocal microscope (Axio-Observer 5; Carl Zeiss, Oberkonchen, Germany).

## 3. Results and Discussion

### 3.1. Synthesis of the BMEDSeDP Crosslinker

A crosslinker is an essential component for designing an appropriate stimulus-responsive system. The chemical bridging of diselenide bonds offers great advantages to be used as a crosslinker because it is easy to oxidize even under mild stimuli due to low bond dissociation energy. In order to shell-crosslink the micelles via DA reaction, we prepared the maleimide-based crosslinker containing diselenide bonds (BMEDSeDP) using HEMI and DSeDPA. Firstly, DSeDPA and HEMI were prepared in two- and three-step reactions, respectively [50,51,52]. Next, the preparation of the crosslinker was performed through the DCC coupling reaction between the hydroxyl group of HEMI and carboxylic group of DSeDPA, and its structure was confirmed by ^1^H-NMR. The maleimide ring proton peaks appeared as a singlet at 6.73 ppm, and methylene protons adjacent to maleimide ring and diselenide appeared as triplets at 4.26 ppm and 2.78 ppm, respectively. Both methylene protons attached to the ester group appeared as triplets at 3.77 ppm and 3.05 ppm.

### 3.2. Synthesis and Characterization of SCL Micelles

**Scheme 2:**
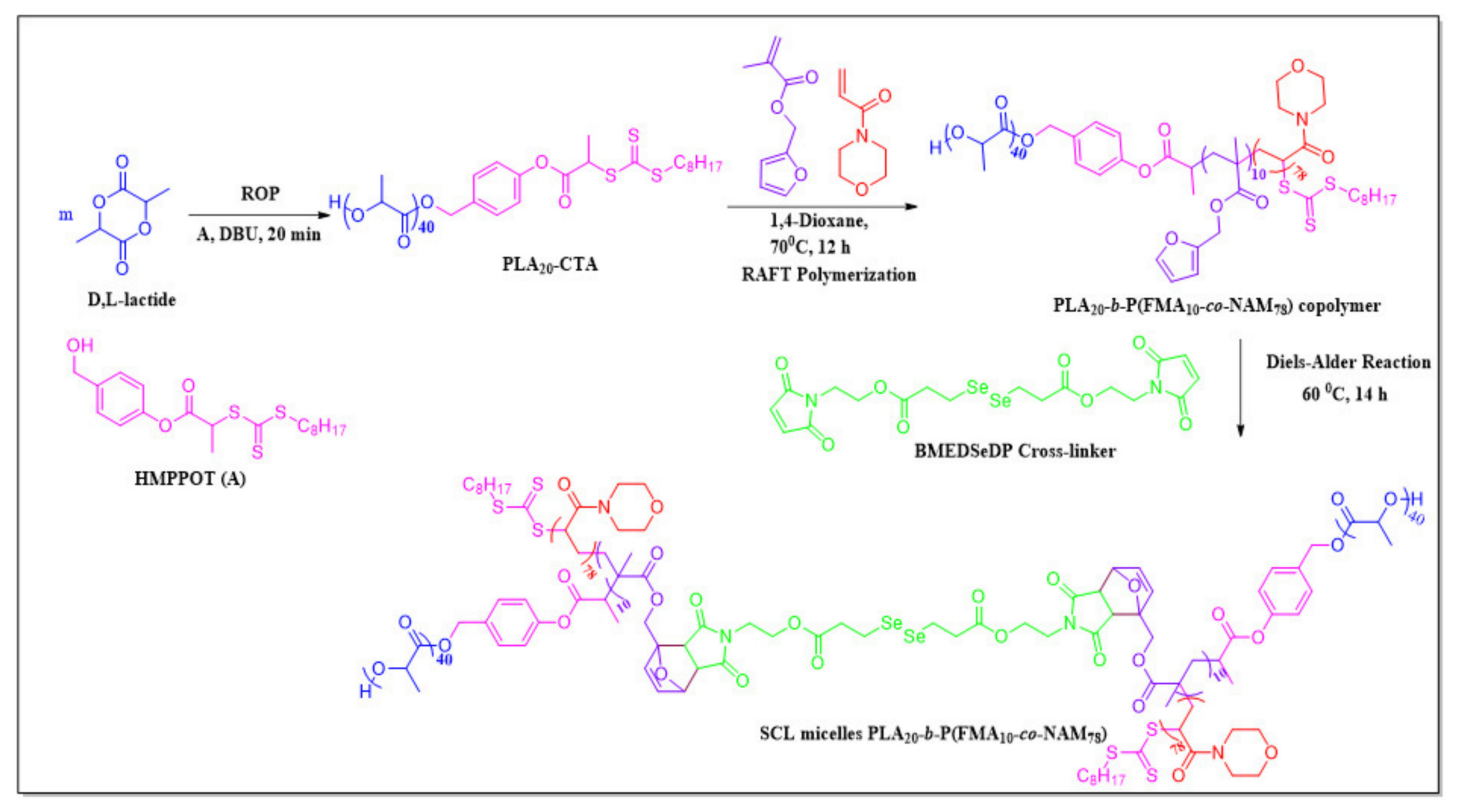
Synthesis of the PLA_20_-*b*-P(FMA_10_-*co*-NAM_78_) copolymer and SCL micelles.

**Figure 1:**
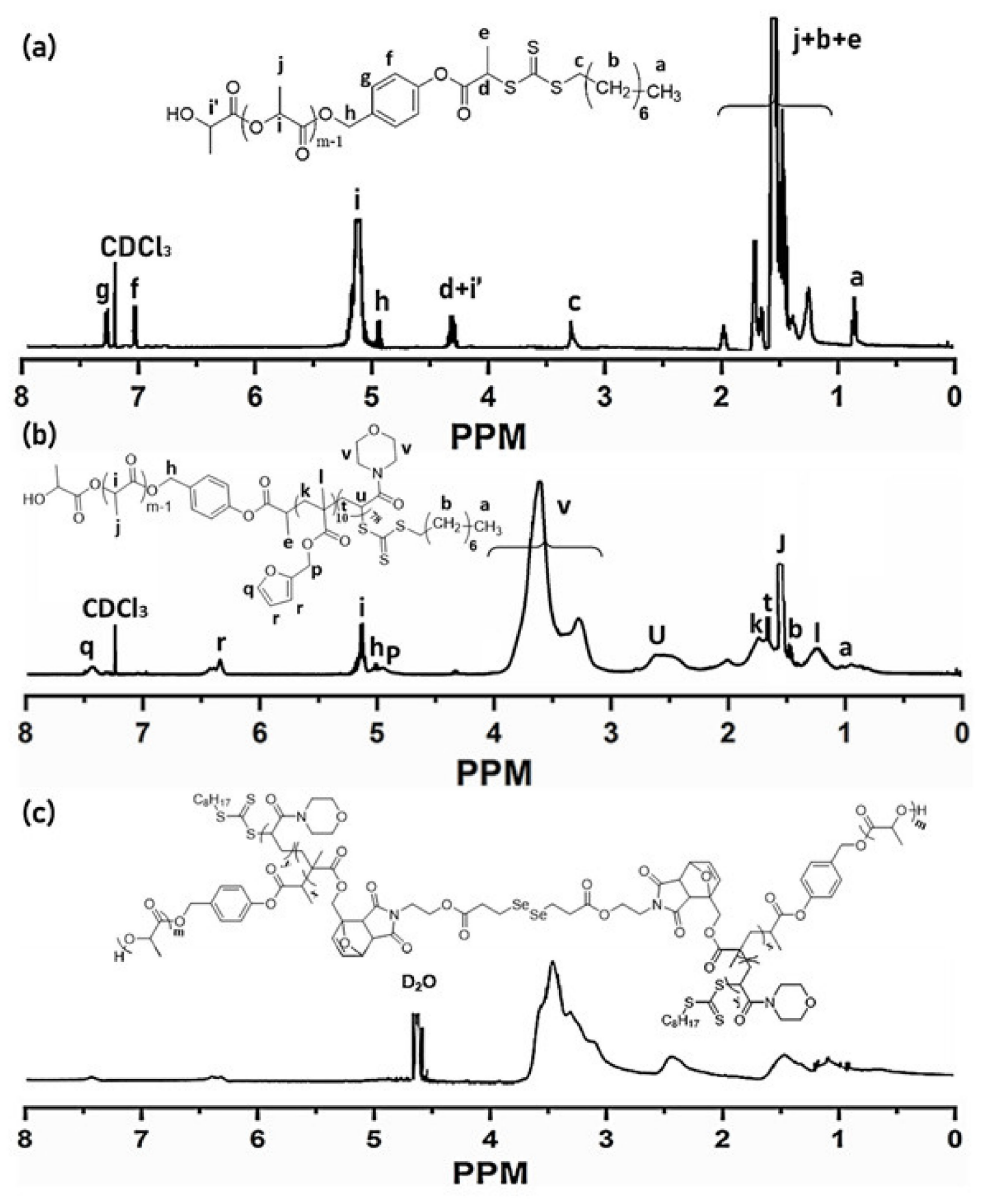
^1^H-NMR spectra of (**a**) PLA_20_-CTA, (**b**) PLA_20_-*b*-P(FMA_10_-*co*-NAM_78_), and (**c**) PLA_20_-*b*-P(FMA_10_-*co*-NAM_78_) SCL micelles.

**Figure 2:**
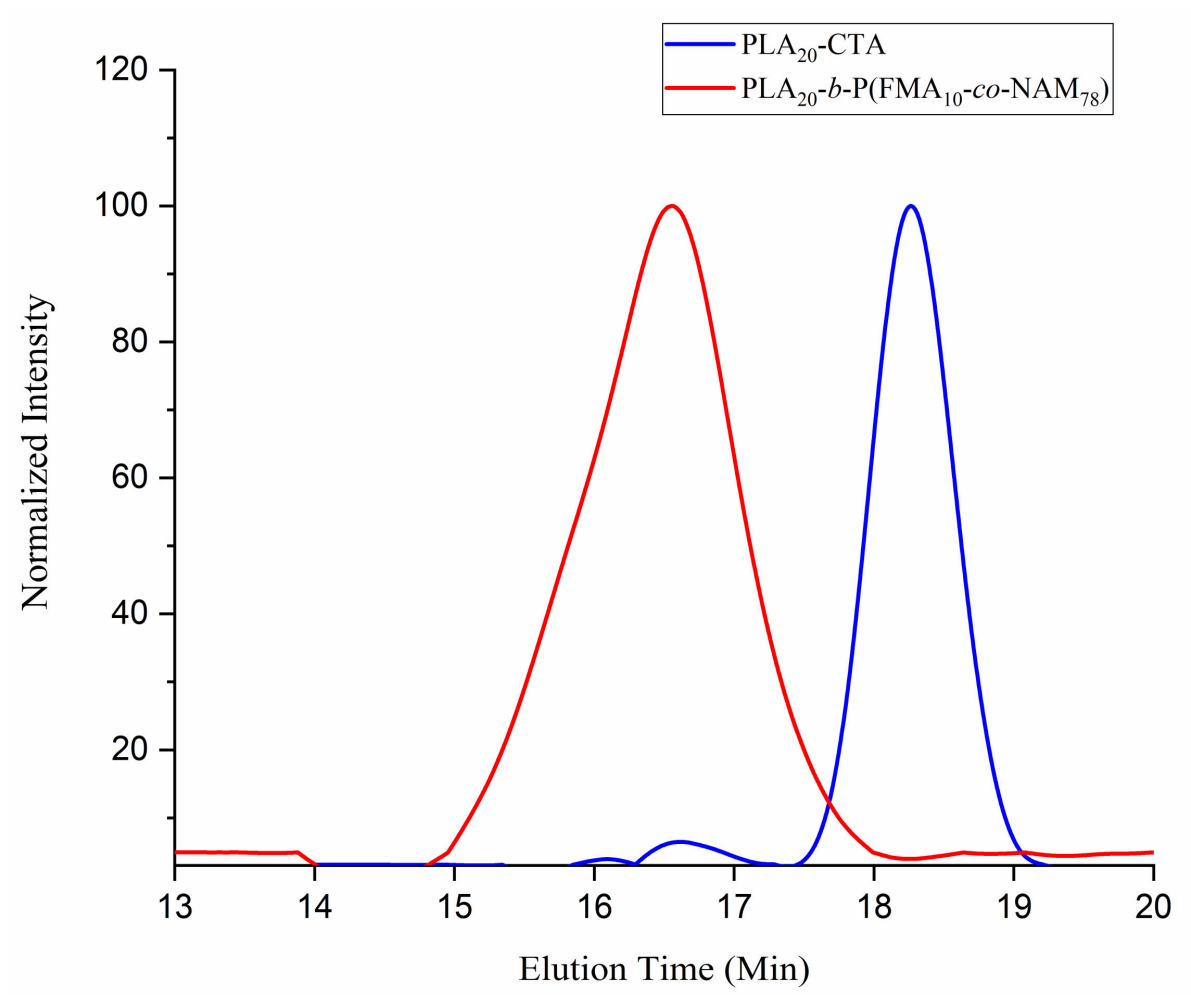
GPC chromatograms of PLA_20_-CTA and PLA_20_-*b*-P(FMA_10_-*co*-NAM_78_).

PLA_20_-*b*-P(FMA_10_-*co*-NAM_78_) was synthesized using RAFT and ROP techniques. The synthetic scheme of PLA_20_-*b*-P(FMA_10_-*co*-NAM_78_) and SCL micelles is shown in Scheme 2. The ROP of LA was performed using the HMPPOT initiator to synthesize PLA_20_-CTA. The ^1^H-NMR spectrum of PLA_20_-CTA (Figure 1a) shows the methyl and methine protons of the RAFT initiator and PLA_20_ moiety at 0.87 ppm and 5.14 ppm, respectively, along with the presence of all the proton peaks of the HMPPOT moiety. The ^1^H-NMR analysis of PLA_20_-CTA confirmed the degree of polymerization (DP) to be about 20 according to the peak integration ratio of methine protons (5.14 ppm) of the PLA_20_ backbone chain and the methyl protons (0.87 ppm) of the RAFT initiator. The molecular weight of PLA_20_-CTA was also determined by GPC analysis. The *M*_n_ of PLA_20_-CTA (*Ð* = 1.13) from GPC analysis was 3400 g/mol, which was comparable to the estimated value of *M*_n_ (3200 g/mol) calculated by ^1^H-NMR (Figure 2, Table 1). PLA_20_-CTA was used as a macro-initiator for the random copolymerization of NAM and FMA in 1, 4-dioxane at 70 °C, resulting in the formation of PLA_20_-*b*-P(FMA_10_-*co*-NAM_78_) copolymer. The ^1^H-NMR spectrum (Figure 1b) of PLA_20_-*b*-P(FMA_10_-*co*-NAM_78_) copolymer shows the methylene protons of PFMA at 5.14 ppm. The protons of the furfuryl ring were observed at 6.36 and 7.44 ppm, while the methylene protons of the PNAM moiety appeared at 3.31–3.96 ppm. The DPs of PFMA and PNAM were confirmed by the peak integration ratios of methine protons (i, 5.14 ppm) of the PLA moiety to furfuryl protons (q, 7.44 ppm) of the PFMA moieties and methylene protons (v, 3.31–3.96 ppm) of PNAM moieties, which were found to be 10 and 78, respectively. The *M*_n_ of PLA_20_-*b*-P(FMA_10_-*co*-NAM_78_) (*Ð* = 1.42) from GPC analysis was 16,400 g/mol, whereas a slightly lower value of *M*_n_ (15,872 g/mol) was observed by ^1^H-NMR (Figure 2, Table 1). SCL micelles were prepared by the DA click reaction between the furfuryl moiety of PLA_20_-*b*-P(FMA_10_-*co*-NAM_78_) copolymer and the maleimide moiety of the BMEDSeDP crosslinker at the shell of the micelles. The ^1^H-NMR (in D_2_O) spectrum of SCL micelles showed that the proton peaks belonging to the PLA hydrophobic cores almost disappeared, whereas the proton peaks corresponding to the hydrophilic part of PNAM and a small amount of PFMA which remained after the DA reaction were still present (Figure 1c). This confirmed that, after shell-crosslinking, the hydrophilic outer shell of PNAM maintained a well-solvated structure.

**Figure 3:**
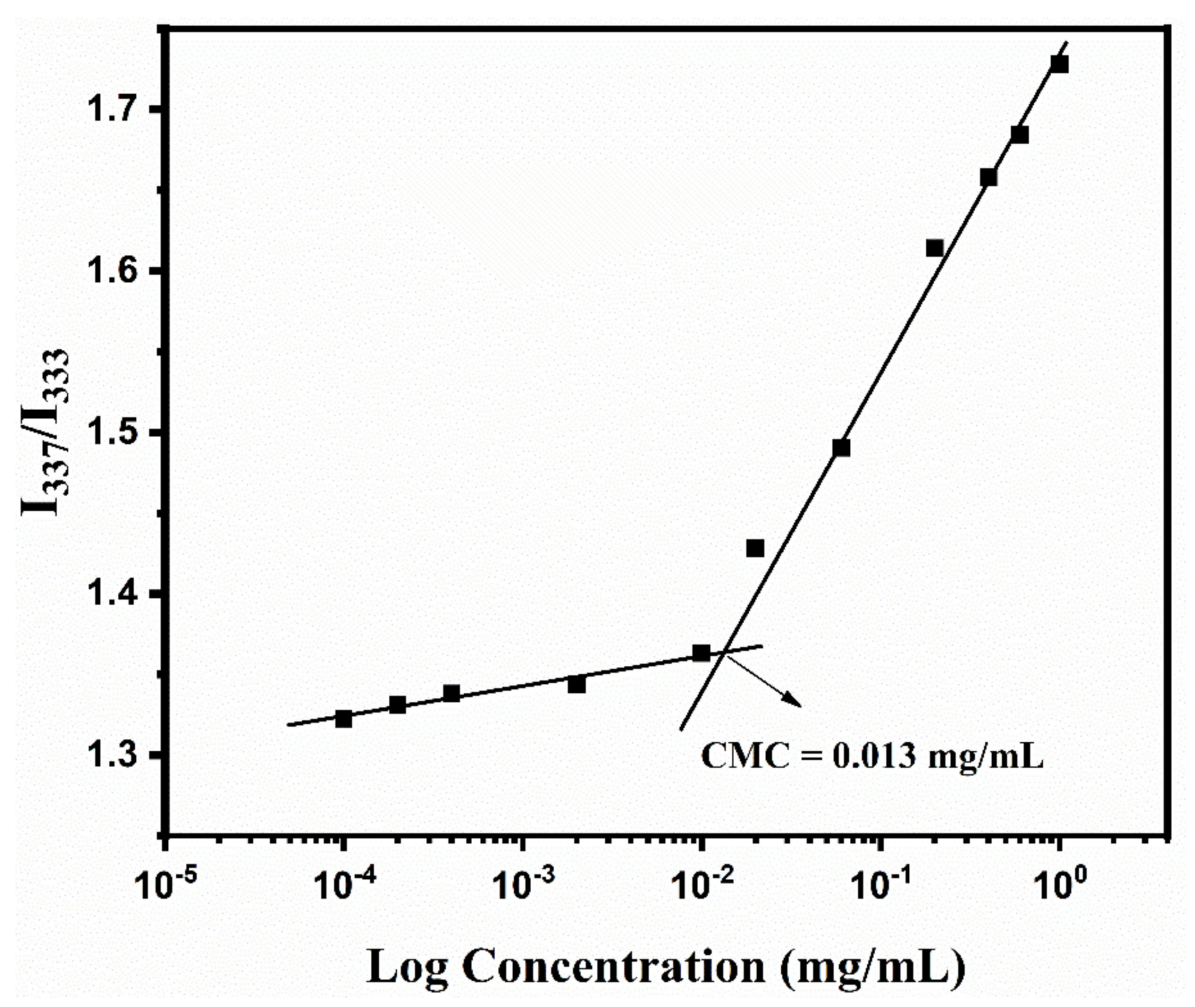
The plot of the pyrene fluorescence excitation intensity ratio (I_337_/I_333_) vs. the function of copolymer concentration.

The CMC of the PLA_20_-*b*-P(FMA_10_-*co*-NAM_78_) copolymer was determined using pyrene as a fluorescence probe. The plot of the pyrene fluorescence excitation intensity ratio (I_337_/I_333_) vs. the function of copolymer concentration (Figure 3) indicated that the CMC of PLA_20_-*b*-P(FMA_10_-*co*-NAM_78_) was 0.013 mg/mL.

**Figure 4:**
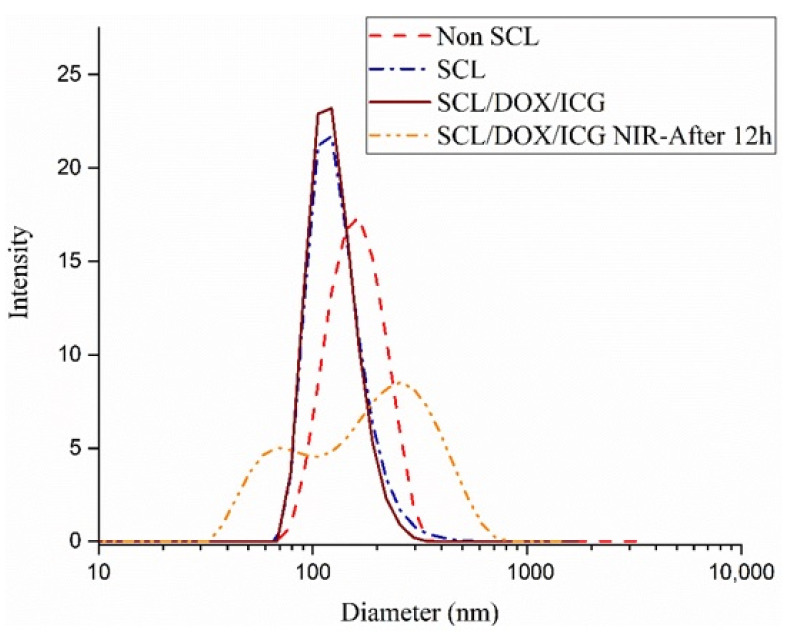
DLS profile of non-SCL, SCL, and SCL/DOX/ICG micelles before and after NIR irradiation.

The hydrodynamic diameters of the self-assembled non-SCL and SCL micelles were measured using DLS techniques (Table 2) and found to be 152 nm and 135 nm, respectively (Figure 4). After the shell-crosslinking, the size of the SCL micelles decreased, which was probably because of shrinkage of the shells caused by the crosslinking reaction. The hydrodynamic diameter of SCL/DOX/ICG micelles slightly increased to 139 nm due to the loading of DOX and ICG. Most notably, the hydrodynamic diameter of SCL/DOX/ICG micelles after 12 h of NIR exposure increased with broad dispersity, indicating the disruption of shell-crosslinking due to the cleavage of diselenide bonds present at the shell of the micelles.

**Figure 5:**
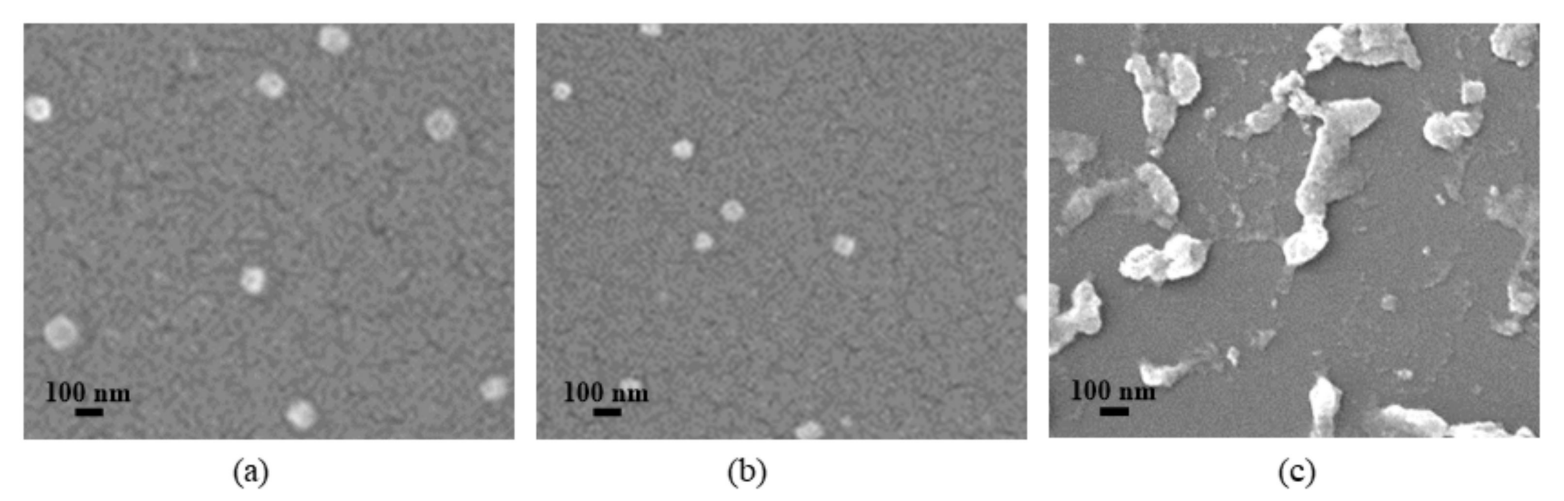
FE-SEM images of (**a**) non-SCL micelles, (**b**) SCL micelles, and (**c**) NIR (5 min)-treated SCL micelles (after 12 h).

FE-SEM images of non-SCL micelles and SCL micelles before and after NIR exposure are presented in Figure 5. Non-SCL and SCL micelles showed a spherical morphology with a smaller average size than the size observed from DLS analysis due to the shrinking of the micellar structure during the sample preparation process. An undetermined size and nonuniform morphology of SCL micelles was observed after 12 h of NIR exposure (5 min) probably due to the decrosslinking of the micellar shells. This result, revealing the degradation of the micellar structure, was consistent with the DLS results.

### 3.3. DOX/ICG-Loading and Characterization of SCL Micelles of PLA_20_-b-P(FMA_10_-co-NAM_78_)

**Figure 6:**
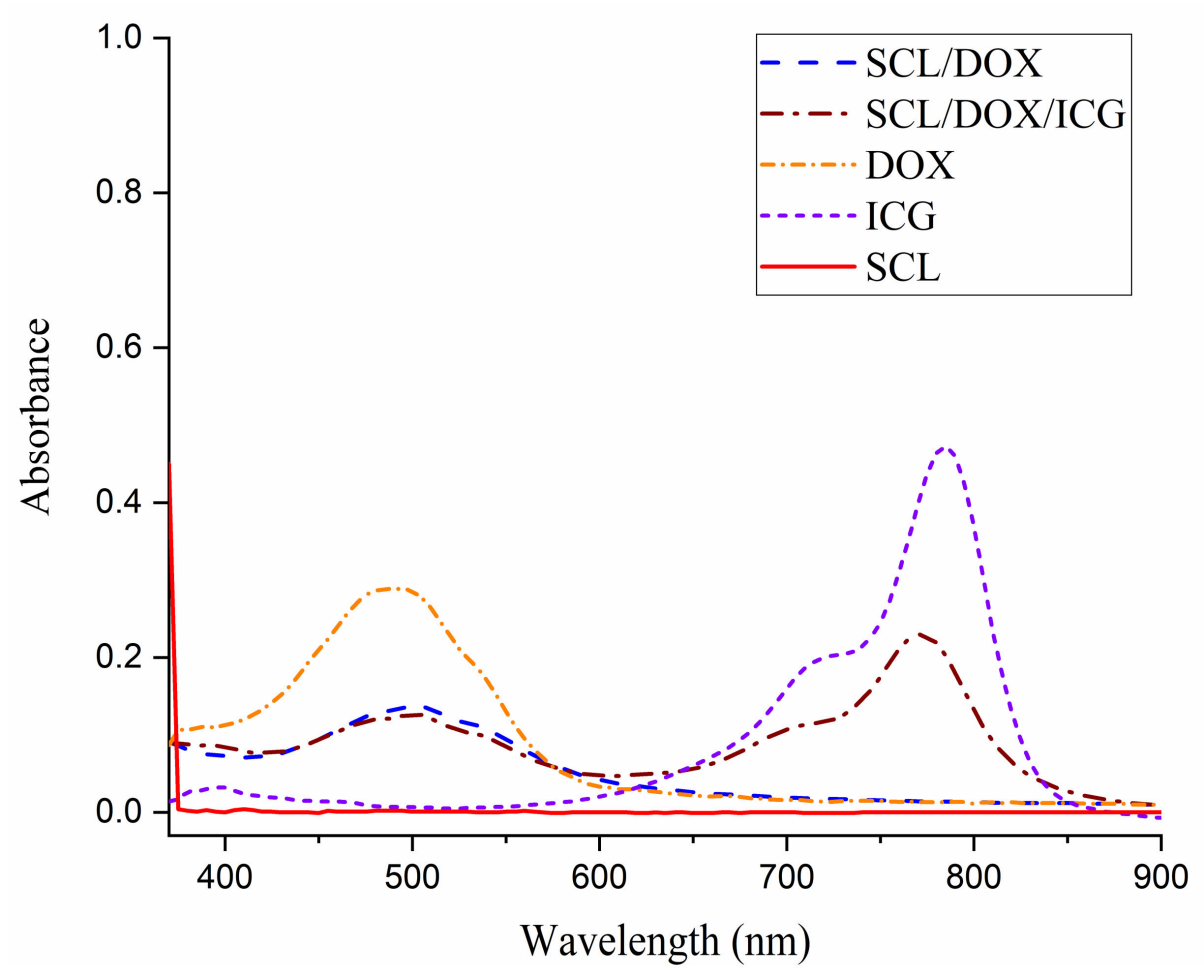
UV/Vis absorption spectra of free DOX, free ICG, SCL, SCL/DOX, and SCL/DOX/ICG.

An anticancer drug, DOX, and NIR-sensitive dye, ICG, were physically encapsulated in the SCL micelles of PLA_20_-*b*-P(FMA_10_-*co*-NAM_78_). The unencapsulated DOX and ICG were removed by centrifugation at 1500 rpm and finally dialyzed for 24 h. The amount of DOX and ICG encapsulated in the SCL micelles was analyzed by a UV/vis spectrophotometer using characteristic absorption peaks at 485 and 780 nm. The characteristic absorption peaks of DOX (485 nm) and ICG (780 nm) exhibited redshifts (505 nm and 805 nm) after loading, demonstrating the successful DOX/ICG encapsulation into the SCL micelles. The LE and LC of DOX into the SCL/DOX micelles were found to be 75.2% and 13.2%, respectively. The hydrophobic interaction of DOX with the micellar core and the pi–pi interaction between DOX and the micellar shell may account for the high DOX-loading ability of the SCL micelles. However, a slight increase in DOX loading was observed when DOX was encapsulated together with ICG, where the LE and LC were found to be 77.1% and 13.5%, respectively (Figure 6 and Table 3). A slight increase in the DOX loading with ICG could be due to the interaction between the negatively charged sulfonate groups in ICG and positively charged amino groups in DOX. Similar result of enhanced DOX encapsulation in the presence of ICG was also observed by Li et al. [53].

### 3.4. NIR-Responsive In Vitro DOX Release

**Figure 7:**
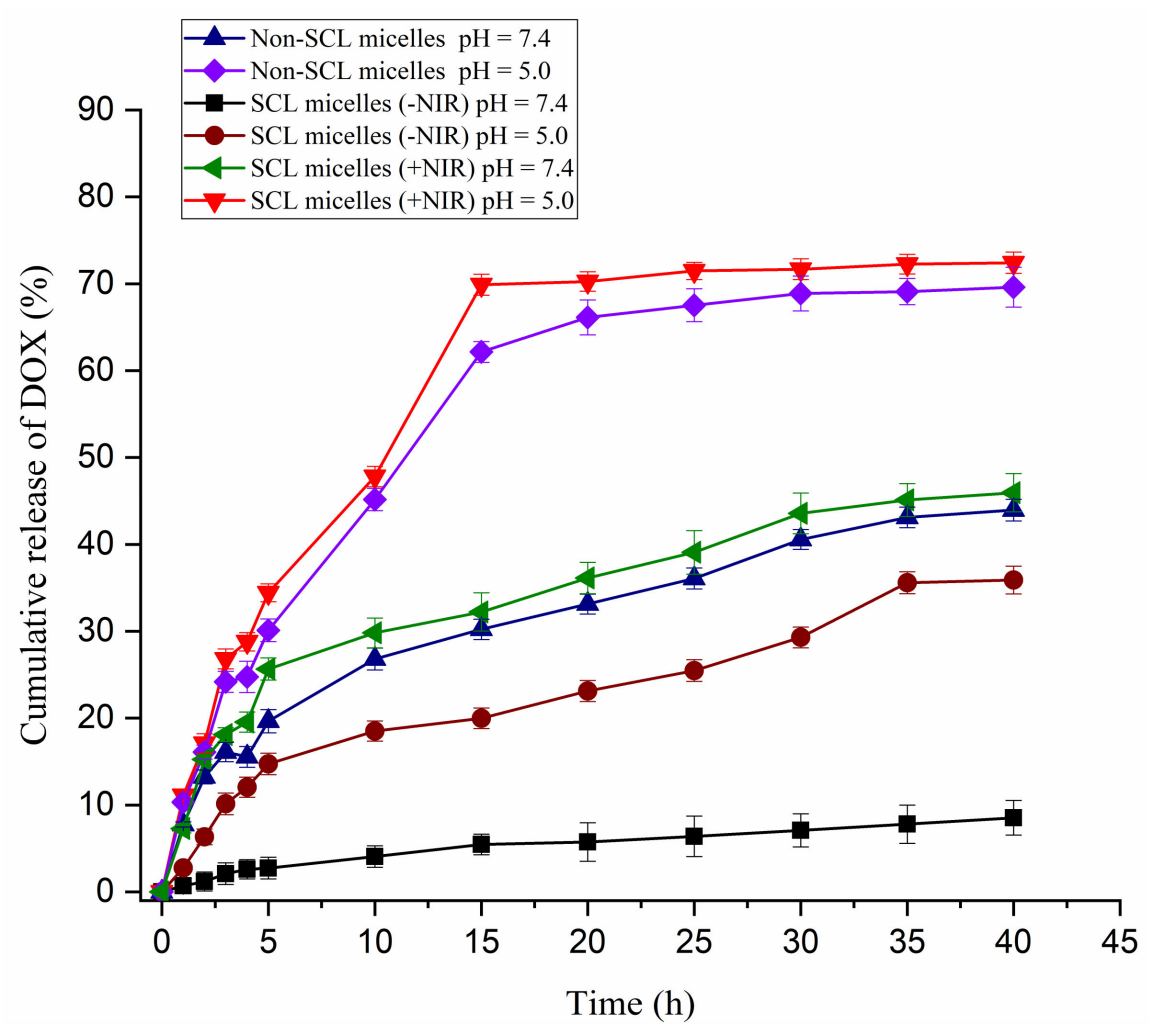
Cumulative release of DOX from non-SCL and SCL micelles at pH 7.4 and pH 5.0 (with and without NIR irradiation for 5 min).

The drug release profile of drug-loaded crosslinked polymeric micelles is an important factor to develop an effective drug delivery system. The drug release behavior of non-SCL micelles was studied along with SCL micelles with or without NIR exposure at pH 7.4 and 5.0 to mimic the physiological environment encountered in normal cells, as well as cancer cells (Figure 7). Under a physiological condition (pH 7.4), SCL micelles and non-SCL micelles exhibited a DOX release of 8% and 43% in 40 h, respectively. The slow DOX release behavior from SCL micelles could be explained by the fact that the shell-crosslinking greatly protects the release of DOX from the micellar structure. This release behavior suggests that SCL micelles have an advantage over non-SCL micelles due to their minimum DOX leakage under physiological conditions (pH 7.4). At pH 5.0 without NIR exposure, enhanced DOX release profiles of SCL micelles (35%) and non-SCL micelles (69%) were observed after 40 h compared with the release at pH 7.4, possibly due to the improved solubility of DOX in acidic conditions [54]. Additionally, due to the compact structure of the shell-crosslinking, the DOX release percentage from SCL micelles was lower than the non-SCL micelles [55]. Under the NIR exposure, the DOX release from SCL micelles increased dramatically under both environments (pH 7.4 and 5.0) compared with the DOX release without NIR exposure. After 40 h, more than twice the drug release (72%) was observed at pH 5.0 under NIR exposure compared to the release without NIR exposure. Moreover, enhanced DOX release was observed at pH 5.0 (72%) compared to pH 7.4 (45%) under NIR exposure. These results validated the role of diselenide bonds in the shell of the micelles, whose cleavage could be controlled by the NIR exposure. The diselenide bond is cleaved by the ROS produced from ICG during NIR exposure, which disrupts the micellar structure to enhance the DOX release [41,56]. The lower release without NIR exposure under similar conditions resulted from the compactness of the SCL structure because the crosslinked micellar coronas act as a diffusion barrier for the drug [20]. Thus, the overall results suggest that SCL micelles release only a negligible amount of encapsulated DOX in the bloodstream (~pH 7.4), whereas a large amount of DOX can be released from the SCL micelles on reaching the cancer cell environment (~pH 5.0) followed by the external NIR exposure.

### 3.5. In Vitro Cytotoxicity and Cellular Uptake of SCL/DOX/ICG Micelles

**Figure 8:**
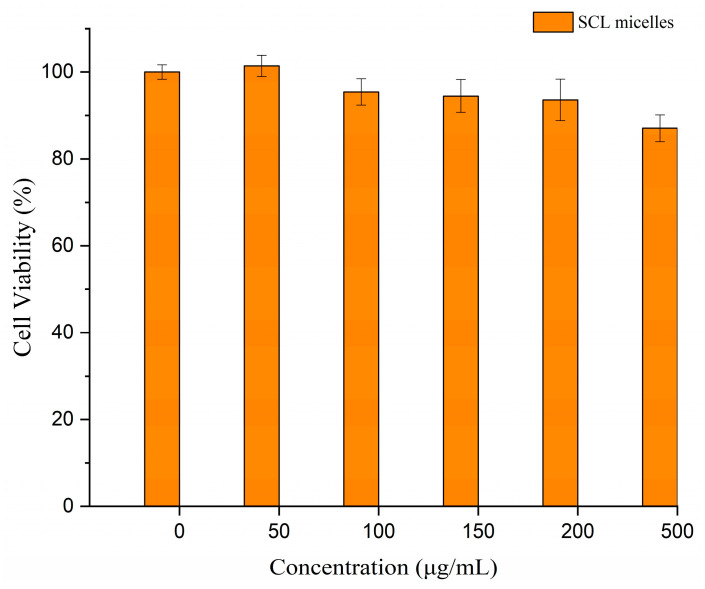
HFF-1 cell viability after 24 h of incubation with SCL micelles.

**Figure 9:**
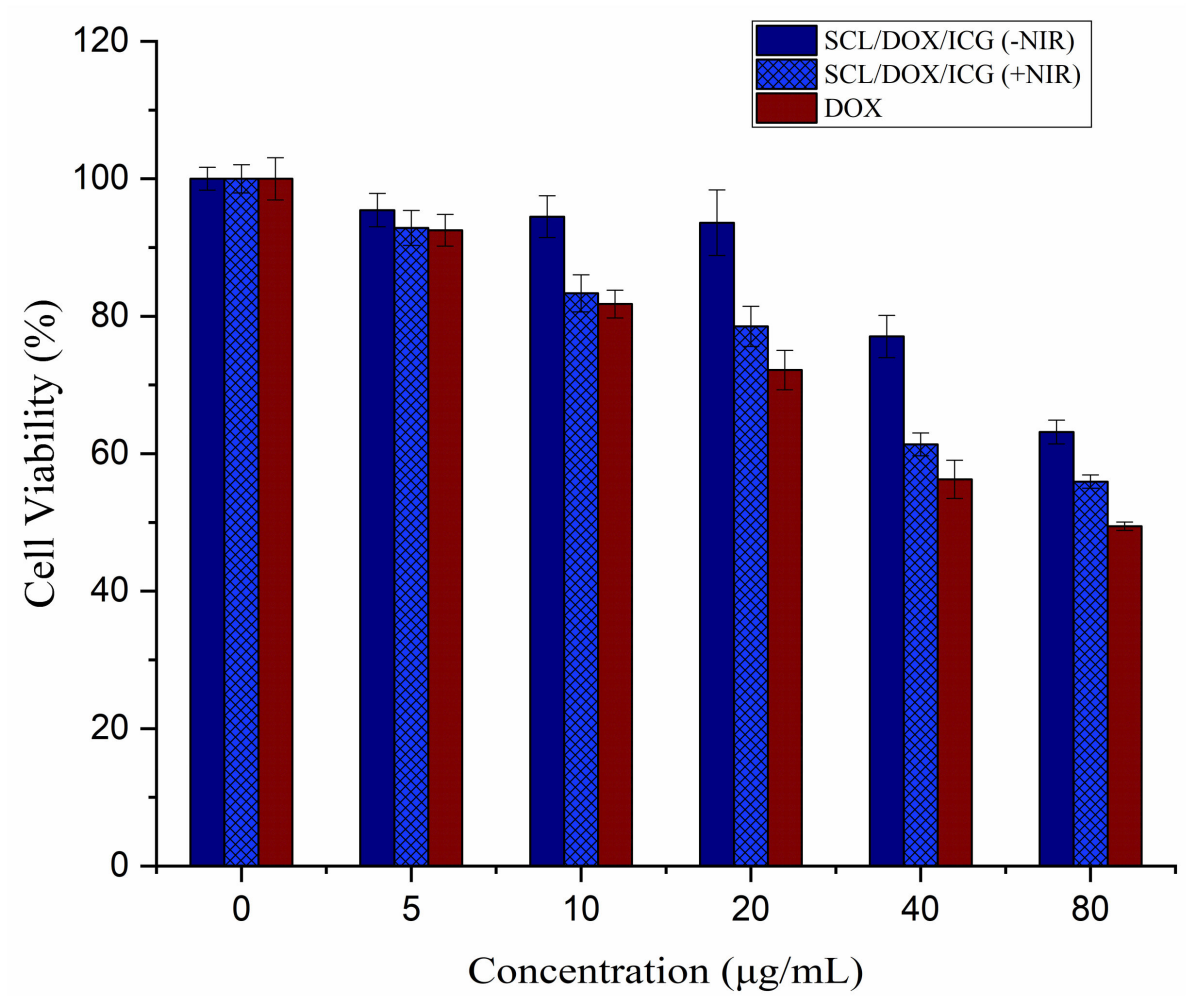
Cell viability of HeLa cells after 24 h treatment with various doses of free DOX and SCL/DOX/ICG micelles with and without NIR (5 min).

HFF-1 cells were used as noncancerous cells to evaluate the biocompatibility of SCL micelles. Blank SCL micelles exhibited more than 85% HFF-1 cell viability on treatment for 24 h up to 500 μg/mL of micelle concentration (Figure 8), indicating good biocompatibility with the normal (HFF-1) cells, presenting their high potential as an effective noncytotoxic delivery vehicle. Furthermore, the cytotoxic effect of the SCL/DOX/ICG micelles was assessed on HeLa cells to evaluate their cancer treatment applicability. SCL/DOX/ICG micelles and free DOX (5–80 μg/mL: DOX concentration) displayed a concentration-dependent decline in HeLa cell viability, signifying the inhibition of the proliferation of cancer cells (Figure 9). The higher cell viability of SCL/DOX/ICG micelles compared to free DOX was possibly due to incomplete release of DOX from SCL/DOX/ICG micelles, which was consistent with the in vitro release study. However, HeLa cell viability of SCL/DOX/ICG micelles without NIR exposure was found to be a little bit higher compared to cell viability with NIR exposure (5 min), indicating the enhanced release of DOX due to the breaking of diselenide bonds from the shells. SCL/DOX/ICG micelles without NIR exposure did not show a significant reduction in cell viability up to 40 μg/mL, while significantly reduced HeLa cell viability was observed at higher concentrations, indicating that NIR irradiation is needed to break the micellar shells for DOX release. The cell viability results indicated that SCL/DOX/ICG micelles induced the HeLa cell apoptosis and exhibited enhanced cell cytotoxicity with NIR exposure.

**Figure 10:**
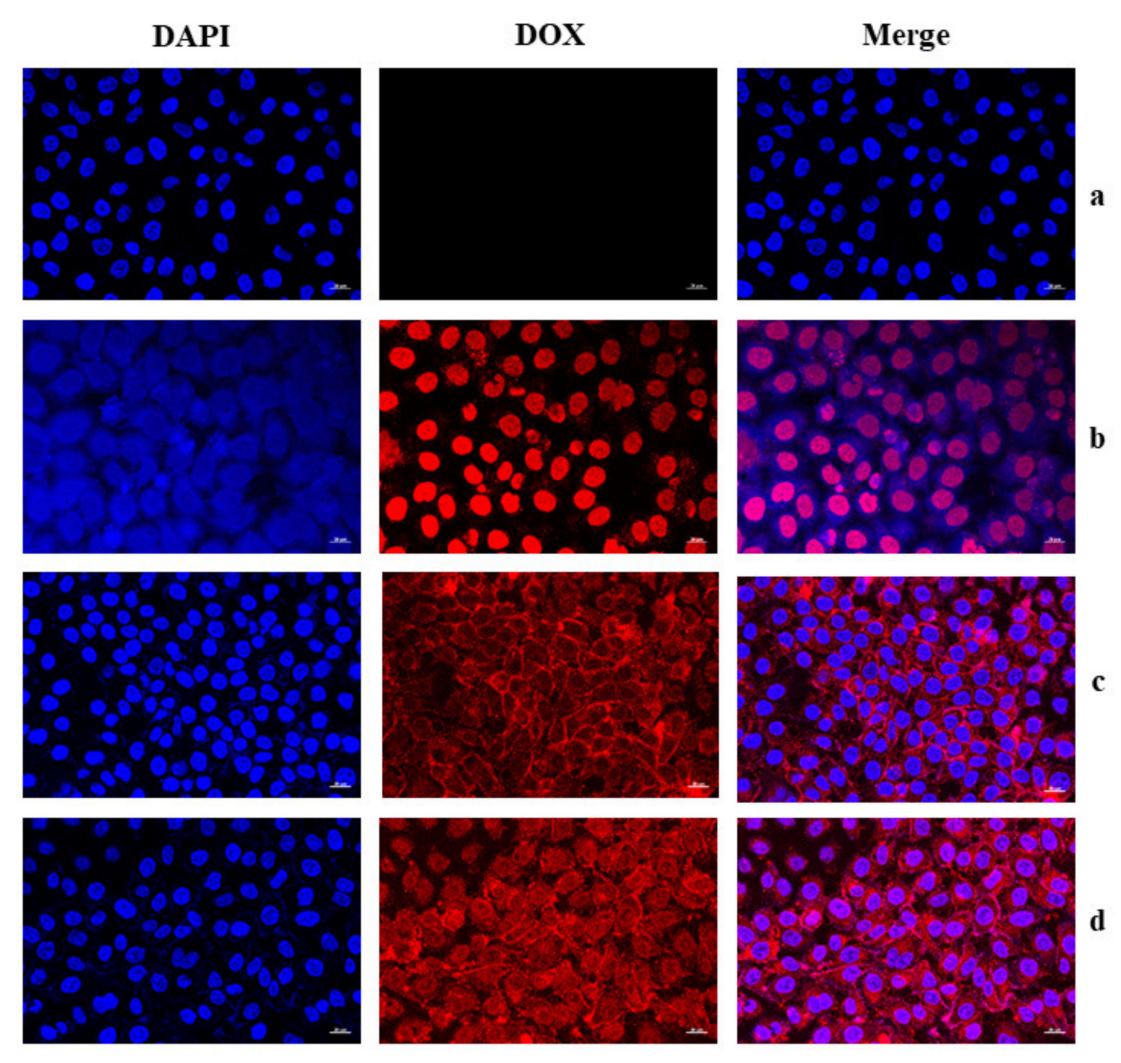
CLSM images of HeLa cells incubated with free DOX and SCL/DOX/ICG micelles (40 μg/mL DOX concentration). For each panel, the images from left to right designate cell nuclei stained with DAPI, DOX fluorescence, and overlays of both (DAPI and DOX) images. Untreated HeLa cells (**a**) and HeLa cells treated with free DOX (**b**) or SCL/DOX/ICG micelles with NIR exposure for 5 min (**c**) and 10 min (**d**).

Cellular uptake of SCL/DOX/ICG micelles and their intracellular DOX release performance were inspected using a confocal laser scanning microscopy. The HeLa cells untreated (panel a, Figure 10) and treated with free DOX (panel b, Figure 10) or SCL/DOX/ICG micelles (DOX concentration: 40 μg/mL) with 5 min (panel c, Figure 10) and 10 min (panel d, Figure 10) of NIR exposure were stained with DAPI. As predicted, no red DOX fluorescence was observed for untreated HeLa cells. Free DOX and SCL/DOX/ICG micelles showed different intracellular distribution of DOX. Free DOX-treated HeLa cells exhibited a strong red fluorescence after 12 h of incubation. On the other hand, weaker red fluorescence was observed in the case of SCL/DOX/ICG micelles compared to free DOX. This is probably due to the reason that SCL/DOX/ICG micelles were internalized by the HeLa cells via endocytosis, and all the DOX could not be released from the micelles [57]. Red fluorescence was observed mostly in the cytoplasm after 5 min of NIR exposure. However, stronger red fluorescence was observed in the cytoplasm and nuclei of the HeLa cells after 10 min of NIR exposure compared to 5 min exposure, indicating the enhanced release of DOX due to the breaking of additional diselenide bonds from the shells. Thus, more DOX could easily internalize the cells and diffuse into the nuclei. The results also demonstrated the NIR-responsive behavior of SCL/DOX/ICG micelles after successful internalization into the HeLa cells to release the DOX in intracellular compartments and induce apoptosis.

## 4. Conclusions

In summary, we designed and synthesized novel SCL micelles of PLA_20_-*b*-P(FMA_10_-*co*-NAM_78_) copolymers. The shells of the micelles containing furfuryl groups were crosslinked using BMEDSeDP crosslinkers via DA reaction. The average sizes and shapes of non-SCL, SCL, and SCL/DOX/ICG micelles were measured by DLS and FE-SEM. DOX encapsulation into SCL micelles slightly increased the micellar size. The SCL micelles of PLA_20_-*b*-P(FMA_10_-*co*-NAM_78_) exhibited a high DOX loading capacity (LE ~77% and LC ~13%). The SCL/DOX/ICG micelles could control DOX release under a physiological environment and release the entrapped DOX rapidly upon NIR exposure owing to decrosslinking of the micellar coronas. The biocompatibility of SCL micelles was verified by their negligible cytotoxicity (>85%) to the normal HFF-1 cells up to the 500 μg/mL of micelle concentration. Furthermore, SCL/DOX/ICG micelles were found to be potent in carrying DOX in the intracellular compartment, triggering apoptosis on HeLa cancer cells upon NIR irradiation. The prepared DOX-encapsulated NIR-responsive PLA_20_-*b*-P(FMA_10_-*co*-NAM_78_)-SCL micelles could be used as a smart nanocarrier for controlled drug release. Further experiments such as in vivo pharmacokinetics and pharmacodynamics studies of PLA_20_-*b*-P(FMA_10_-*co*-NAM_78_)-based SCL micelles are necessary to realize their application and will be done in the future.

## Figures and Tables

**Table 1:** Characteristic data of PLA_20_-CTA and PLA_20_-*b*-P(FMA_10_-*co*-NAM_78_).

Run	Polymer	Mn (NMR) ^a^ (g/mol)	Mn (GPC) ^b^ (g/mol)	*Đ*
1	PLA_20_-CTA	3200	3400	1.13
2	PLA_20_-*b*-P(FMA_10_-*co*-NAM_78_)	15,872	16,400	1.42

^a^ Determined by ^1^H-NMR. ^b^ Determined by GPC (THF, 1 mL/min, 25 °C) calibrated against PS standards.

**Table 2:** Size and PDI of the non-SCL, SCL, and SCL/DOX/ICG micelles.

Samples	Size (DLS)	PDI
Non-SCL	152 ± 45	0.258
SCL	135 ± 31	0.293
SCL/DOX/ICG	139 ± 31	0.295

**Table 3:** DOX and ICG encapsulation in SCL micelles.

Samples	LE of DOX (%)	LC of DOX (%)
SCL/DOX	75.2%	13.2%
SCL/DOX/ICG	77.1%	13.5%

## Data Availability

The data presented in this study are available upon request from the corresponding author.
